# Supplementary material for: Editing of endogenous tubulins reveals varying effects of tubulin posttranslational modifications on axonal growth and regeneration
Source: eLife. 2024 Jul 1;13:RP94583. doi: 10.7554/eLife.94583 (PMC11216746; doi:10.7554/eLife.94583)

Figure 3E raw image

Tubulins	+	+	-	+	-	+	+
ATP	-	+	+	+	+	+	+
Kinase	-	-	CDK1	CDK1	MBK-2	MBK-2	MBK-2
Buffer	MEM	MEM	MEM	MEM	MEM	MEM	BRB80

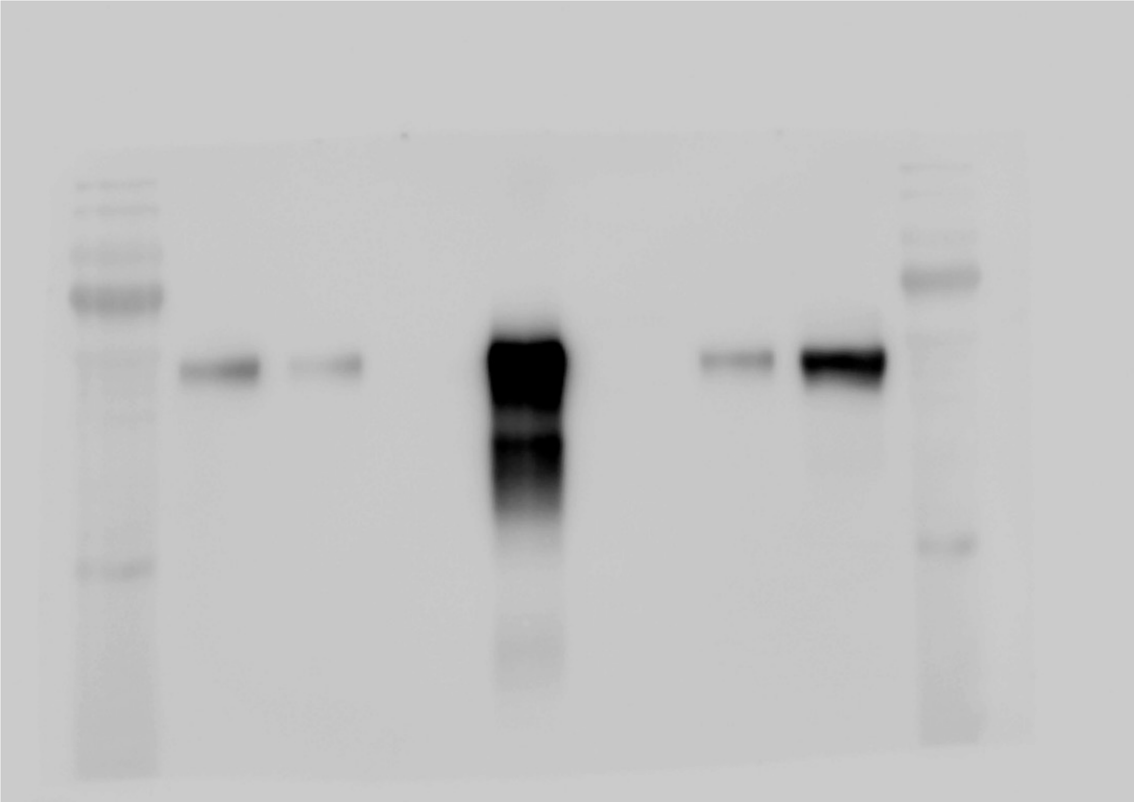

Anti-phospho-S172 tubulin

Tubulins	+	+	-	+	-	+	+
ATP	-	+	+	+	+	+	+
Kinase	-	-	CDK1	CDK1	MBK-2	MBK-2	MBK-2
Buffer	MEM	MEM	MEM	MEM	MEM	MEM	BRB80

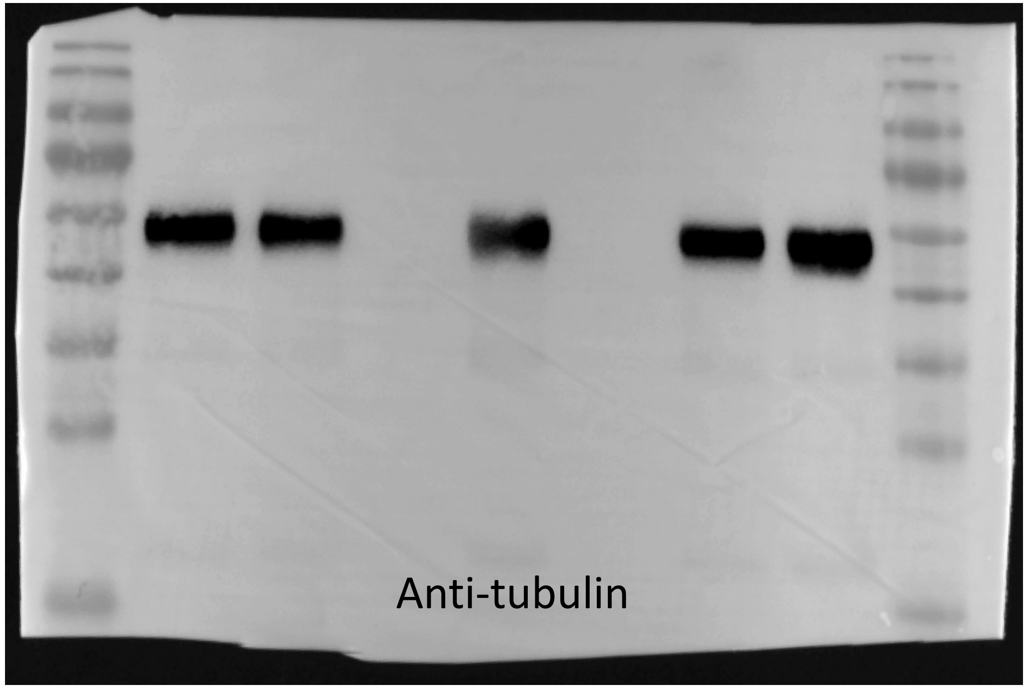

Anti-tubulin

Figure 3 – figure supplement 3 raw image

Tubulins	-	+	+	+	+	+
ATP	+	+	+	+	+	+
MBK-2 (nM)	1742	0	800	1268	1742	1742
Buffer	BRB 80	BRB 80	BRB 80	BRB 80	BRB 80	MEM

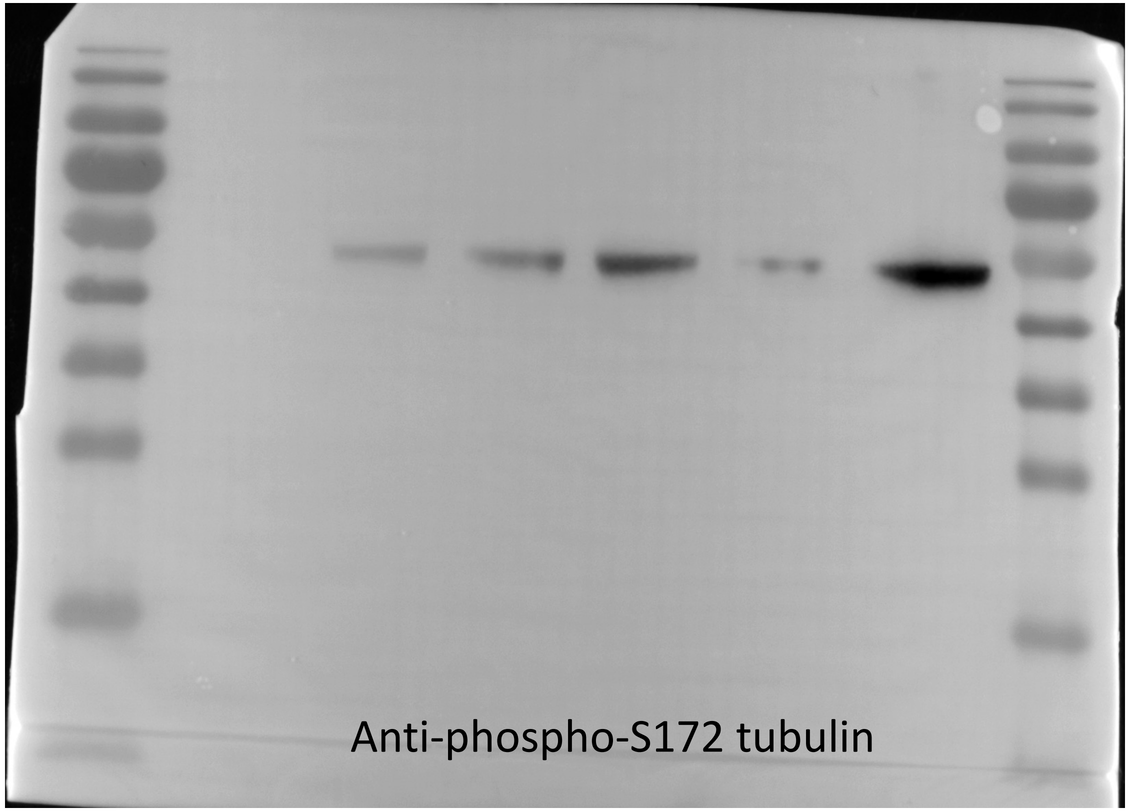

Supplement: Figure 3—source data 2. [file elife-94583-fig3-data2.pdf]
