# Supplementary figures and images for: Editing of endogenous tubulins reveals varying effects of tubulin posttranslational modifications on axonal growth and regeneration
Source: eLife. 2024 Jul 1;13:RP94583. doi: 10.7554/eLife.94583 (PMC11216746; doi:10.7554/eLife.94583)

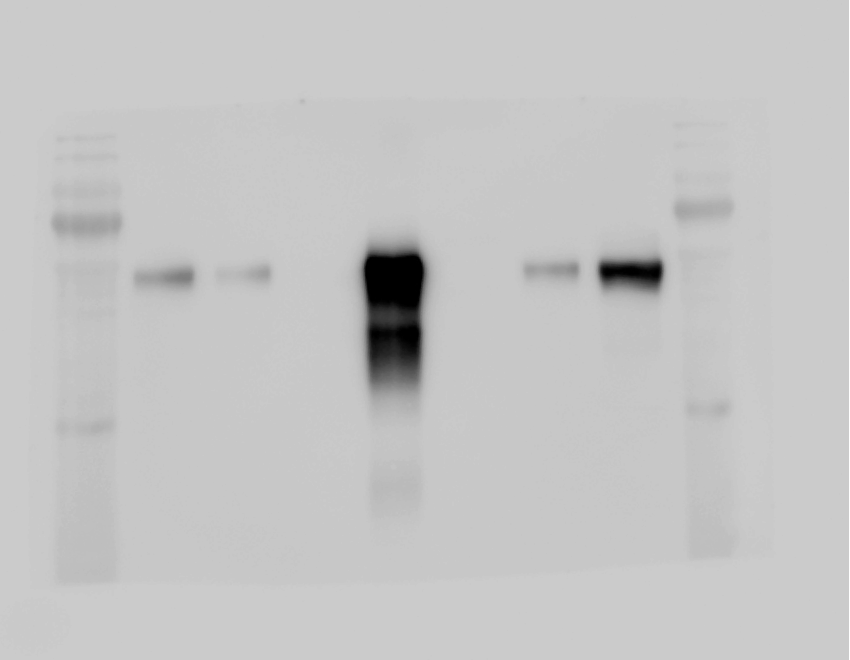

Supplement: Figure 3—source data 3. [file elife-94583-fig3-data3.zip › Western blot/Picture1.png]

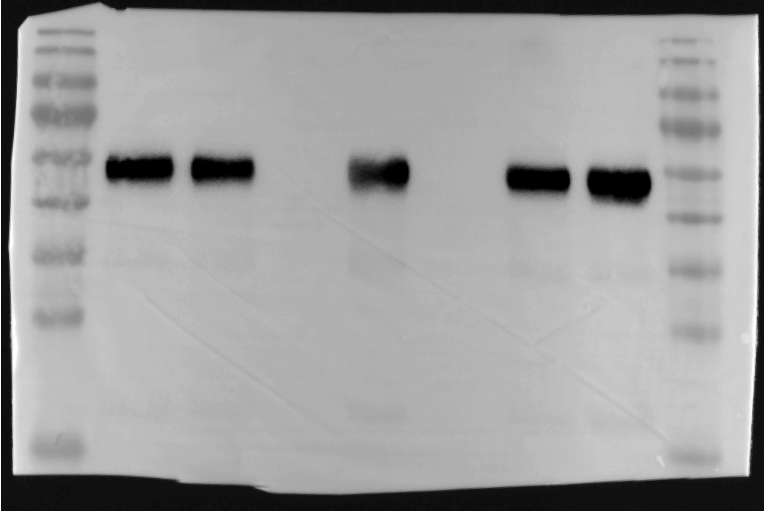

Supplement: Figure 3—source data 3. [file elife-94583-fig3-data3.zip › Western blot/Picture2.png]

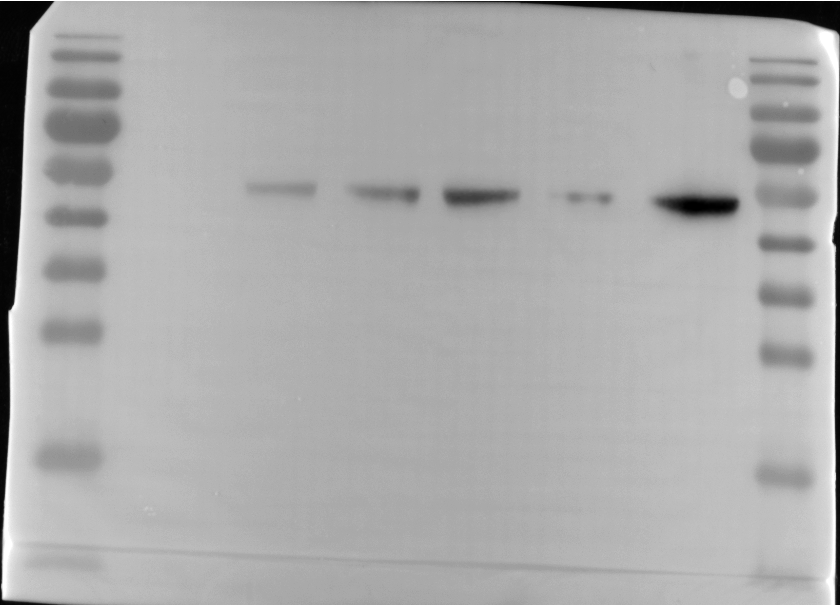

Supplement: Figure 3—source data 3. [file elife-94583-fig3-data3.zip › Western blot/Picture3.png]
